# Impact of Metformin and Pioglitazone on Serum Level of Tumor Necrosis Factor-Alpha and Lipid Profiles during Implantation Window in Diabetic Rats

**DOI:** 10.22074/ijfs.2019.5636

**Published:** 2019-04-27

**Authors:** Abbas Bakhteyari, Parvaneh Nikpour, Fatemah Sadat Mostafavi, Nahid Eskandari, Mohammad Matinfar, Sara Soleimani Asl, Roshanak Aboutorabi

**Affiliations:** 1 Department of Anatomical Sciences, Faculty of Medicine, Isfahan University of Medical Sciences, Isfahan, Iran; 2 Department of Genetics and Molecular Biology, Faculty of Medicine, Isfahan University of Medical Sciences, Isfahan, Iran; 3 Child Growth and Development Research Center, Research Institute for Primordial Prevention of Non-communicable Disease, Isfahan University of Medical Sciences, Isfahan, Iran; 4 Department of Immunology, Faculty of Medicine, Isfahan University of Medical Sciences, Isfahan, Iran; 5 Department of Internal Medicine Faculty of Medicine, Isfahan University of Medical Sciences, Isfahan, Iran; 6 Endometrium and Endometriosis Research Center, Hamedan University of Medical Sciences, Hamedan, Iran

**Keywords:** Diabetes Mellitus, Embryo Implantation, Metformin, Pioglitazone, Tumor Necrosis Factor-Alpha

## Abstract

**Background:**

The present study was designed to evaluate serum lipid profile and tumor necrosis factor-alpha (TNF-ɑ) level in diabetic rats at implantation time. Type 2 diabetes mellitus (T2DM) could affect various systems, including innate immune system and it causes chronic low-grade inflammation, increasing level of TNF-ɑ. Furthermore, T2DM is often accompanied by impaired lipid profile. Metformin and pioglitazone are used as the first and second lines of treatment for T2DM.

**Materials and Methods:**

In this experimental study, 35 adult virgin female wistar rats, weighting 175-225 g, were randomly categorized into five groups: i. Control, ii. Sham, iii. Nicotinamide (NA)+streptozotocin (STZ) induced T2DM, iv. Diabetic+pioglitazone (20 mg/kg/day for 28 days oral administration), and v. Diabetic+metformin (100 mg/kg/day for 28 days oral administration). At the time of implantation, TNF-ɑ level in serum of rats was measured by ELISA kit. Glucose was measured using photometric method and lipid profiles were calculated by enzymatic methods.

**Results:**

Level of TNF-ɑ in the diabetic group was significantly higher than other groups (P<0.001). In metformin treated group, TNF-ɑ serum level was also significantly higher than pioglitazone treated group (P<0.001). Fasting blood sugar (FBS) and lipid profiles were significantly higher in diabetic group.

**Conclusion:**

Metformin and pioglitazone have similar effects on glucose, lipid profiles and TNF-ɑ serum levels. Among these drugs, pioglitazone has more efficient influence on TNF-α serum level, in comparison with metformin.

## Introduction

Type 2 diabetes mellitus (T2DM), especially while it is not well controlled, can affect various systems including the innate immune system, and cause chronic low-grade inflammation in the body (1, 2). In addition, diabetes also affects the functions of female reproductive system and occurrence of subfertility (3) or fetal loss after implantation in diabetic women is more than healthy individuals (4). Different functions of female reproductive system, such as the menstruation, pregnancy, ovulation and implantation, are affected by several hormones and various inflammatory cytokines such as tumor necrosis factoralpha (TNF-α), interleukin-6 (IL-6) and IL-1 (5).

Studies suggest that hormone-based disorders and high levels of inflammatory cytokines can lead to interruptions in the immune-endocrine cross talk within endometrium, myometrium and blastocyst that could interferes trophoblast and decidua interaction during pregnancy (6, 7). Furthermore, increased TNF-α production is related to infertility and recurrent spontaneous abortion, but the issue is open to further discuss (8). Localized inflammation improves the implantation outcomes and it has positive relationship with cytokine expressions, such as TNF-α, in endometrial biopsies (9, 10). Therefore, optimal expression of TNF-α could be useful during pre-implantation and implantation periods (11).

Up to now, several drugs are available for the treatment of T2DM, through which, biguanides (metformin) and thiazolidinedione (pioglitazone) were used in this study. Metformin is used as the first line treatment to reduce serum level of glucose in diabetic patients (12). It has been reported that the aforementioned drug plays a crucial role in modulating inflammatory cytokine levels and lipid profile (13, 14).

Pioglitazone is a member of the thiazolidinedione family which binds to the peroxisome proliferator-activated receptor gamma (PPAR-γ), increasing insulin sensitivity, improving lipid profile in serum and regulating blood pressure (15). Pioglitazone also reduces TNF-α level in serum (16).

Therefore, the aim of this study was to determine TNF-ɑ levels and lipid profile in serum of diabetic rat models after treatment with metformin and pioglitazone during embryo implantation window period.

## Materials and Methods

### Animal maintenance

This study was an experimental study on diabetic rat models, conducted at the central laboratory of Isfahan University of Medical Sciences (Isfahan, Iran) in 2018. All experimental procedures were approved by Isfahan University of Medical Sciences Animal Ethical Committee (code number IR.MUI. REC.1394.1.184.). Adult virgin female Wistar rats, weighting 175-225 g and aged 6-8 weeks, were purchased from Pasteur Institute of Iran (Tehran, Iran), maintained in conventional wire mesh cages at room temperature regulated at 21 ± 1°C, humidity 45-50%, and 12 hours light/dark cycle, while they were accessed to standard dry pellets and water.

### Induction of diabetes

Nicotinamide (NA) and streptozotocin (STZ, both from Sigma-Aldrich, Germany) were used to induce T2DM in animals. First, NA with 200-230 mg/kg dose was injected intraperitoneally (IP). After 15 minutes, STZ was IP injected with dose of 60 mg/kg (17). Three days after T2DM induction, blood samples were taken to measure blood glucose in animals using a glucometer (HemoCue Glucose 201+, Sweden). If fasting blood sugar (FBS) level was higher than 250 mg/dl, it was considered as diabetic rat (18).

### Study design and serum collection

Thirty-five rats were randomly divided into five groups as follows, existing seven rats in each group. Control group, sham group that received just normal saline using IP injection, STZ+NA-induced diabetic group without any treatment (FBS ≥250 mg/dl), diabetic groups which received pioglitazone 20 mg/kg/day for 28 days by orogastric gavage (19) and the last group was diabetic rats which received metformin 100 mg/kg/day for 28 days by orogastric gavage (20).

**Fig 1:**
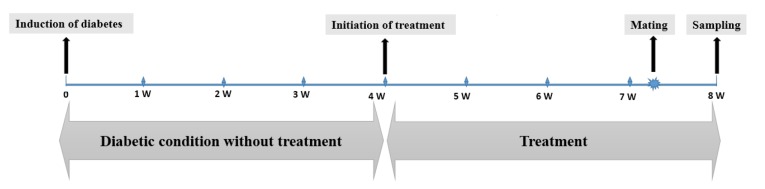
The study was designed for 8 weeks, at the end of week 4, after induction of diabetes, administration of drugs were started. Mating was occurred at the 8th week and sampling was performed 4 days later.

Animals were maintained in diabetic condition for four weeks and then drug therapy was started for the next 4 weeks as shown in Figure 1. FBS levels were measured every 4 days by glucometer (HemoCue Glucose 201+, Sweden) and droplet samples were collected from dorsal vein of tail.

In the 4^th^ week, twenty-four days after administration of metformin or pioglitazone, two females and one male rat, in all groups, were placed in the separate cages for mating. The next day, the female rats were checked for the presence or absence of vaginal plugs. Presence of the vaginal plug revealed the first day of pregnancy and the time of implantation window was considered 4 days after that (21), which means 28^th^ day after beginning of the treatment. Four weeks after treatment with metformin or pioglitazone, at the time of blastocyst implantation in rats, animals were fasted overnight and sacrificed by injection of ketamine hydrochloride (50 mg/kg) and xylazine hydrochloride (7 mg/ kg) (IP). Blood samples were taken by cardiac puncture then placed in tubes at room temperature for 30 minutes and allowed to get clotted. Aforementioned samples were centrifuged at 3000 rpm for 10 minutes. Serum was removed and stored at -20 ºC until biochemical analysis.

### Determination of TNF-ɑ, lipid profile and glucose

Serum level of TNF-ɑ was measured by enzyme-linked immunosorbent assay (ELISA) using readymade kit reagents supplied by Eastbiopharm, China (22). Serum glucose level was measured using photometric method and lipid profiles, including triglyceride (TG), cholesterol (Chol), low density lipoprotein (LDL) and high density lipoprotein (HDL) were calculated by enzymatic methods. Measurements were performed by 14000 auto-analyzer (Toshiba, Japan) using manual colorimetric method (23).

### Statistical analysis

Statistical analyses were performed using SPSS version 20.0 (SPSS Inc., USA). The differences were compared using one-way analysis of variance (ANOVA), following Tukey post hoc test for TNF-α and lipid profiles in all groups. P<0.05 was statistically considered significant difference.

## Results

### Pioglitazone and metformin decreased glucose level in the treated rats

**Fig 2:**
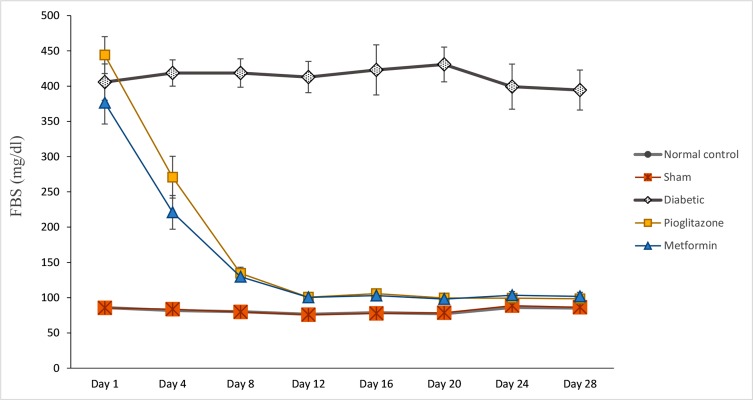
Effects of pioglitazone and metformin on blood glucose level compared to STZ+NA induced diabetic rat models during 4 weeks treatment. FBS was measured every 4 days, during 4 weeks treatment. All values were presented as mean ± standard error mean (mean ± SEM) and there are seven rats in each group. STZ; Streptozotocin, NA; Nicotinamide, and FBS; Fasting blood sugar.

Glucose levels were measured every 4 days, in all groups, until 28^th^ day. As Figure 2 illustrates, both of pioglitazone and metformin regulated blood glucose level after 8 days administration. On the twelfth day, blood glucose reached to normal level and there was no significant difference between normal control group compared to pioglitazone treated group (P=0.103) and metformin treated group (P=0.105).

**Fig 3:**
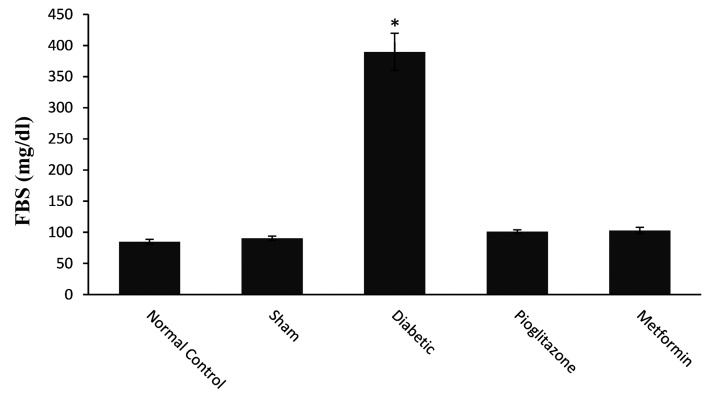
Effects of pioglitazone and metformin, at the time of blastocyst implantation on serum glucose level of rats, compared to STZ+NA induced diabetic rat models at the 28th day of treatment. All values are presented as mean ± SEM. Significant differences in FBS level between diabetic group and all of the other groups were observed (P<0.001). *; Shows significant difference, STZ; Streptozotocin, NA; Nicotinamide, and FBS; Fasting blood sugar.

 Serum glucose levels were significantly higher (P=0.000) in the diabetic group than others control and treatment groups (Fig .3). As shown in Figure 3, metformin and pioglitazone reduced blood glucose levels; so that, there was no significant difference in blood glucose levels between the treated groups compared to normal control group (P=0.363 and P=0.410, respectively). Furthermore, there was no significant difference in blood glucose levels between these two treated groups (P=0.910).

### Pioglitazone and metformin decreased lipid profiles

**Fig 4:**
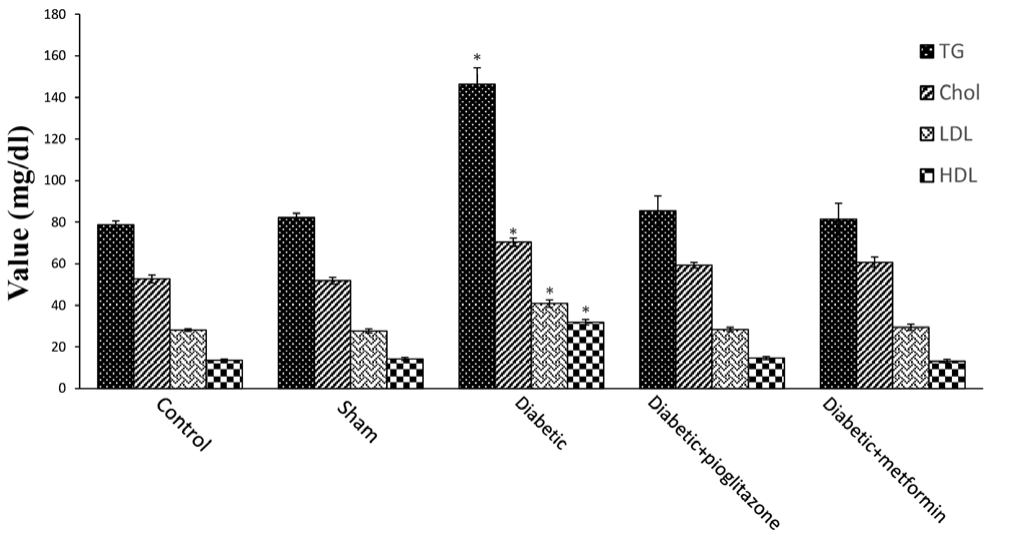
Effect of pioglitazone and metformin, at the time of blastocyst implantation, on lipid profiles in serum of diabetic rats in all five groups. Data are represented as mean ± SEM. *; P≤0.05 vs. control. Lipid profile levels include: TG, Chol, LDL and HDL, measured in 28th day after initiation of treatment. *; Shows significant difference in lipid profile level between diabetic group and all of the other groups (P<0.001), TG; Triglyceride, Chol; Cholesterol, HDL; High density lipoprotein, and LDL; Low density lipoprotein.

Based on our statistical analysis performed on lipid profiles at the end of study, the diabetic group had higher serum TG (P=0.000), Chol (P=0.000), HDL (P=0.000) and LDL (P=0.000) levels than the control, sham and treated groups (Fig .4). There was no considerable difference in lipid profiles, including TG (P=0.643), Chol (P=0.597), HDL (P=0.571), LDL (P=0.281), between two treated groups.

### Increased TNF-α in the diabetic group and decreased TNF-α after treatment

**Fig 5:**
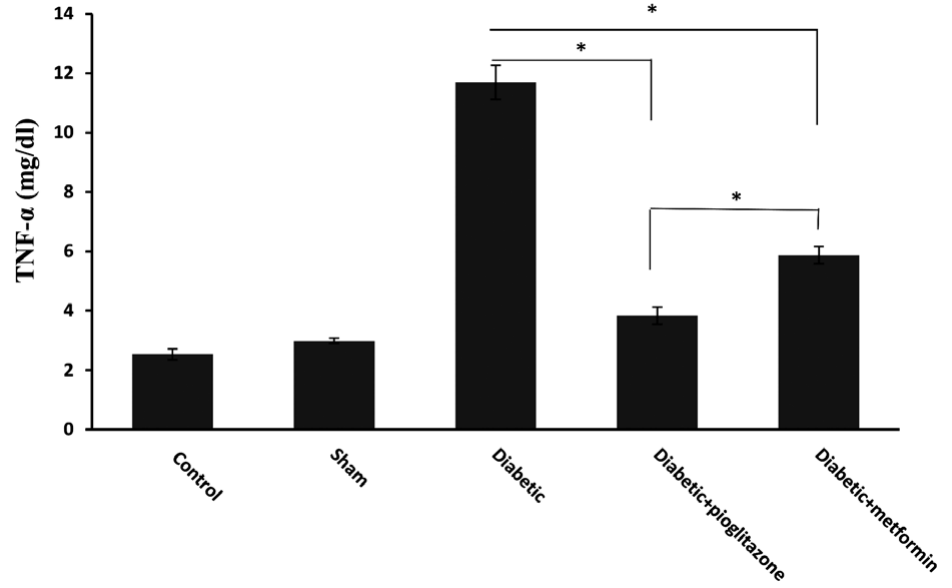
TNF-α serum concentration of T2DM rats treated with metformin and pioglitazone, 4 weeks after treatment. Pioglitazone and metformin significantly decreased TNF-α level compared to diabetic group (P<0.001). A significant difference between treated pioglitazone and metformin was observed (P<0.001). *; Shows significant difference in TNF-α serum levels, TNF-α; Tumor necrosis factor-alpha, and T2DM; Type 2 diabetes mellitus.

Measurement of TNF-α showed that there was no significant difference between sham and control groups (P=0.335). In addition, there is no significant difference between pioglitazone and sham treated groups (P=0.075). However, our results revealed a meaningful difference between pioglitazone and the control group (P=0.008), because TNF-α level in sham group is higher than control, despite not being statistically significant (P=0.335). Based on the ELISA outcomes of TNF-α levels, there was a significant difference between the diabetic group and all of the other studied groups (Fig .5). Moreover, there was a significant difference between the two treated groups with pioglitazone and metformin (P=0.000), and the level of TNF-α in the pioglitazone treated group was significantly lower than the metformin treated group.

## Discussion

Diabetes mellitus, as a long term metabolic perturbation, could lead to reduction of life quality in the affected population as well as increases morbidity, mortality and complications in patients (24). Statistics indicated that global outbreak of the diabetes mellitus is increasing and is going to be a serious problem in the health care debates around the world (25).

According to our study, at the time of rat blastocyst implantation, day 4 of post-coitum, serum glucose level in diabetic group was higher and as expected, significantly different from all other groups. In addition, significant differences were again observed in lipid profile between diabetic group and the other four groups. After 28 days treatment by metformin and pioglitazone, serum glucose level was normalized in the treated groups. Along with the improvement of blood glucose levels, both drugs had an appropriate impression on lipid profiles (i.e. the levels of TG, Chol, LDL and HDL). Our study revealed that TG, Chol, LDL and HDL levels were significantly raised in the diabetic group. Regarding the literature reviews, HDL should be reduced in T2DM (26-28).

Considering the impression of changes in female hormones, especially estrogen, through pregnancy -from implantation to child birth- HDL level was increased (29-31). On the other hand, in early stage of T2DM, HDL serum level is significantly higher than control group (32, 33). Lawrence et al. (26) performed a study on diabetic patients and compared the effect of metformin, pioglitazone and gliclazide on lipid profile. They concluded that there is no significant difference in lipid profile before and after administration of these drugs. With regards to administration of pioglitazone in diabetic patients, Aghamohammadzadeh et al. (28) mentioned that pioglitazone down-regulates FBS, hemoglobin A1C (HbA1c) and TG levels significantly, but no significant reduction was observed in cholesterol, LDL and HDL levels. These results were not in accordance with our findings. It seems that performing these human studies, without considering life styles, were probably the cause of controversy in variation of lipid profile outcomes. Exercise, individual diet and BMI in each patient could lead to bias in the study. On the other hand in animal experiments, the confounding factors, such as age, sex, exercise and dietary programs, weight, circadian cycle and etc., were taken under precise controlled condition (34).

 The other objective, in our study, was to compare the effects of metformin and pioglitazone on the serum level of TNF-α, as a member of pre-inflammatory cytokine family. T2DM, as an inflammatory condition, could elevate various inflammatory serum cytokines, such as TNF-α, which increase the subsequent complications of this disease (7). The pathogenesis of 10-20% of infertile cases is related to higher level of serum immunological factors, compared to fertile individuals (35). IL-2 and TNF-α cytokine increases could have negative impression on successful pregnancy. When these two cytokines were injected into pregnant mice, the carriages were terminated (36). However, cytokines also exert beneficial effects on pregnancy, including resistance to intrauterine infections and important roles in angiogenesis and tissue regeneration (37). Therefore, to achieve normal and healthy pregnancy, appropriate and adequate presence of pre-inflammatory cytokines in the endometrium has high necessity.

In our present study, level of TNF-α was raised in the serum of diabetic rat model at the time of implantation window, while this level was reduced in rats treated with any of both drugs. Interestingly, our study revealed that the influence of pioglitazone on TNF-α level was significantly more efficient than metformin.

Pioglitazone can reduce serum TNF-α level by several mechanisms, including inhibition of TNF-α production from macrophages (38), suppression of TNF-α mRNA expression from subcutaneous adipose tissue (16), reduction of the number of CD3+ T lymphocytes in diabetic rats, producing higher levels of TNF-α and IL-1β (39).

Embryo implantation is a multifactorial phenomenon which involves precise molecular programming. Accurate duration of existence and efficient levels of the molecular elements depends on healthy and normal metabolism. Diabetes mellitus, as one of the most common metabolic disorders, makes deep disturbances in the levels of molecular production and the period of their presence (40). Therefore, there is an important necessity to pay attention to underlying metabolic diseases and their effects on the fate of natural and assisted pregnancies.

## Conclusion

In this study, at the time of implantation window (i.e. blastocyst-endometrium dialog period), T2DM increases glucose and lipid profile as well as the serum level of TNF-α. Regulation of these parameters was observed after administration of each of metformin and pioglitazone. Additionally, our study reveals that pioglitazone has significantly more efficient influence on TNF-α serum level in comparison with metformin.
